# Biophysical control of plasticity and patterning in regeneration and cancer

**DOI:** 10.1007/s00018-023-05054-6

**Published:** 2023-12-15

**Authors:** Nirosha J. Murugan, Solsa Cariba, Sawith Abeygunawardena, Nicolas Rouleau, Samantha L. Payne

**Affiliations:** 1 Department of Health Sciences, Wilfrid Laurier University, Waterloo, ON Canada; 2 Allen Discovery Center, Tufts University, Medford, MA USA; 3 Department of Biomedical Engineering, Tufts University, Medford, MA USA; 4 Department of Biomedical Sciences, Ontario Veterinary College, University of Guelph, Guelph, ON Canada

**Keywords:** Plasticity, Patterning, Biophysics, Cancer, Regeneration

## Abstract

Cells and tissues display a remarkable range of plasticity and tissue-patterning activities that are emergent of complex signaling dynamics within their microenvironments. These properties, which when operating normally guide embryogenesis and regeneration, become highly disordered in diseases such as cancer. While morphogens and other molecular factors help determine the shapes of tissues and their patterned cellular organization, the parallel contributions of biophysical control mechanisms must be considered to accurately predict and model important processes such as growth, maturation, injury, repair, and senescence. We now know that mechanical, optical, electric, and electromagnetic signals are integral to cellular plasticity and tissue patterning. Because biophysical modalities underly interactions between cells and their extracellular matrices, including cell cycle, metabolism, migration, and differentiation, their applications as tuning dials for regenerative and anti-cancer therapies are being rapidly exploited. Despite this, the importance of cellular communication through biophysical signaling remains disproportionately underrepresented in the literature. Here, we provide a review of biophysical signaling modalities and known mechanisms that initiate, modulate, or inhibit plasticity and tissue patterning in models of regeneration and cancer. We also discuss current approaches in biomedical engineering that harness biophysical control mechanisms to model, characterize, diagnose, and treat disease states.

## Introduction

Organisms achieve and maintain multicellularity by promoting cooperation and mediating conflict within groups of cells, thus prioritizing the collective over its units [1]. How do multicellular systems determine which individual cells should proliferate, specialize, or die in service of the group? This challenge is best illustrated by ontogenetic development, which involves the transition from a single cell to a unified organism comprising billions or trillions of cells. Embryonic cells must display sufficient plasticity to generate, prune, and remodel dozens of specialized tissues during morphogenesis before suppressing these same mechanisms to achieve stable, long-term maintenance of form [2]. However, even the cells of mature organisms retain the potential to re-active latent plasticity and patterning programs to repair or regenerate damaged tissues while suppressing spontaneous and disordered growth including cancers [3–5]. Indeed, cancer and regeneration are related physiological processes with similar levels of plasticity and markedly different capacities to pattern cells into cooperative tissue structures [6–8]. While biomolecular controls of plasticity and patterning are frequently discussed in the literature in the context of cellular communication, less attention has been afforded to biophysical controls including mechanical, electrical, magnetic, and optical signals. Here, we provide a review of known biophysical control mechanisms of tissue plasticity and patterning with a focus on regeneration and cancer as representative model systems. We examine mechanical, optical, electrical, and magnetic signaling modalities as parallel communication channels within tissue microenvironments and their roles as determinants of cell state and fate.

### Tissue plasticity

Plasticity is a property of cells that enables phenotypic changes without genetic mutation and is typically activated as a response to signals and cues within tissue microenvironments [4, 9, 10]. Embryonic organisms display significant plasticity, enabling groups of cells to reconfigure bodies with segments, compartments, layers, topologies, and pigmentation patterns. Unlike humans, organisms such as axolotls can re-activate latent developmental pathways to regrow appendages and re-pattern disorganized tissues [11]. This remarkable state of tissue plasticity is termed “regeneration”. To replace lost or damaged cells, a pool of progenitors must be amassed, which may involve altering the epigenetic state of resident cells [12]. These progenitors, while initially possessing a relatively high degree of plasticity, must then cease proliferation and differentiate into the required somatic cell types for tissue replacement. Cellular plasticity is, therefore, an important element of regenerative success [13]. Despite retaining the same genes that regulate development throughout the lifecycle, only a limited set of mature human tissues retain their intrinsic plasticity, including adipose tissue, connective tissue, and to a much lesser extent, neural tissues [14, 15]. Consequently, humans suffer significantly and often permanently from lacerations, burns, limb loss, degenerative diseases, and other morbidities.

**Fig. 1:**
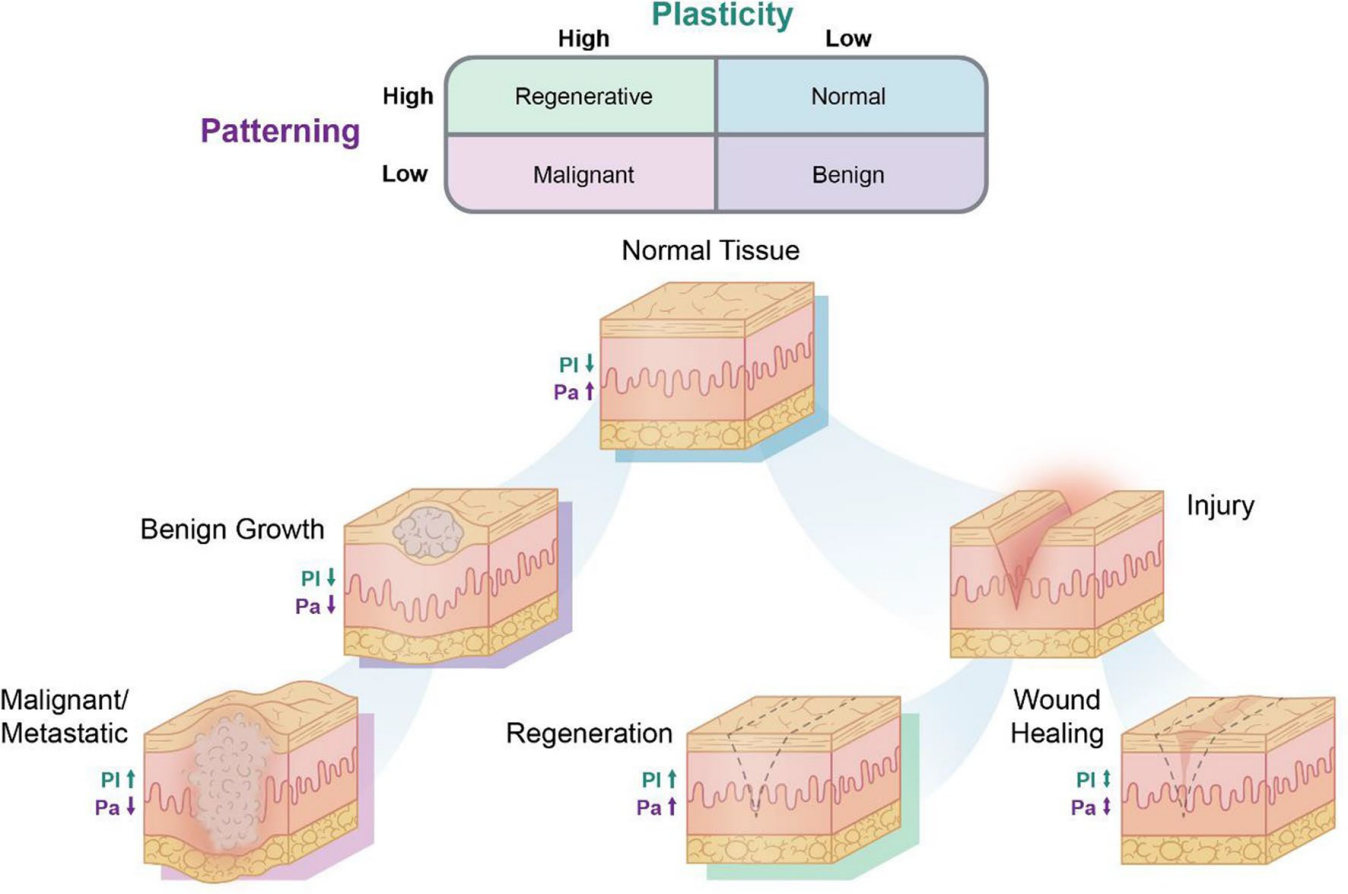
Tissue states differ across dimensions of plasticity and patterning. A two-dimensional model of tissue state with low or high levels of plasticity and patterning accommodates tumor formation and response to injury. Normal, healthy tissues display high degrees of patterning with low levels of plasticity. As patterning decreases, tissues become disorganized with benign growths that can become malignant and metastatic as plasticity increases. Similarly, following an injury, tissue expressing high degrees of plasticity and patterning can re-establish pre-injury phenotypes; however, if these processes are not sufficiently promoted, wounds heal imperfectly with indelible fibrotic scar tissue.

When cellular plasticity subverts key safeguards and becomes unrestrained, as is the case with cancer, tissue architecture can become irreversibly unstable as cells proliferate uncontrollably, invade foreign tissues, and contribute to widespread dysfunction and increased mortality [16]. The suppression of pro-regenerative pathways may have been selected due to their physiological overlap with tumorigenesis [17, 18], which threatens stable multicellularity. Indeed, maintaining low levels of plasticity would greatly favor the long-term structural stability of the organism’s cellular collective over the immortality of individual cancer cells [19]. Interestingly, when cancer cells are introduced into the microenvironments of embryonic or regenerating tissues, they become assimilated and differentiate into stable, somatic cells, suggesting that the microenvironment contains key regulatory signals that control plasticity [20–22]. What types of signals cause multicellular systems to “switch” their plasticity toward embryonic, regenerative, and carcinogenic states (Fig. 1)? Understanding how plasticity is activated or suppressed is integral to the development of regenerative therapies with significant clinical applications for dementia, stroke, spinal cord repair, heart disease, and limb loss.

### Tissue patterning

Tissue patterning involves the integration of repeatable elements within the structure of an organism, including pigmentation and body segmentation. Originally predicted by Turing [23], the discovery that spatial patterning in biochemical systems including tissues can spontaneously emerge from the diffusion of molecules and related interactions was a paradigm-shifting achievement in the life sciences. Indeed, we now know that living systems derive their unparalleled complexity from simple, repeatable events at the sub-cellular level [24, 25], forming repeatable segments, internal compartments, and unique topologies. However, without control mechanisms with which to guide patterning, multicellularity can suffer from inadequate coordination and ultimately collapse [26, 27]. Fortunately, cells and their combinatory structures provide all the necessary conditions to initiate and maintain stable patterning at multiple scales. They generate internal and external gradients, isolate electrochemical reactions within internal compartments, sense microenvironmental cues, self-destruct upon losing control of their basic functions, and communicate with each other using a suite of signals. Indeed, communication is central to multicellular life [28, 29], enabling the continuous flow of information between cells and within changing tissue microenvironments to orchestrate local and long-range tissue dynamics, including plasticity and patterning. While molecular signals including morphogens, hormones, and neurotransmitters are clear determinants of cell fate and behavior toward tissue patterning in both regeneration and cancer (e.g., Notch, Wingless-related integration site (WNT), etc.) [30], mounting evidence suggests that biophysical signals serve as equally important top-down regulators of tissue patterning that can interact and synergize with molecular pathways [31–34]. For example, gene expression can be induced by physically deforming cells such as fibroblasts and cardiomyocytes, which can in turn alter their structure and the biochemical composition of the surrounding microenvironment, thus modifying the impact of subsequent physical stimuli on the tissue, its cell population, and the surrounding extracellular matrix (ECM) [35].

### Regeneration and cancer

Cancer is defined by its highly plastic state and disorganized patterning, imperfectly mirroring normal morphogenetic processes [36–38]. Some authors have suggested that cancer is fundamentally a developmental disorder characterized by a failure to suppress latent plasticity and maintain stable tissue patterns [39, 40]. The parallels between cellular processes in regeneration and cancer have long been recognized [6, 7] and the two states share many common signaling mechanisms [8]. Many of the mechanisms essential to drive the cellular plasticity required for regeneration are co-opted to promote neoplasia and cancer progression. For example, the microRNA miR-21 mediates downregulation of growth-suppressing proteins in healing tissues in the mammalian brain, skin, and liver, as well as limb and kidney regeneration in fish and salamanders [41]. It is also associated with neoplasia, permitting unchecked cell proliferation [42], and is elevated in heterogeneous tumors which are associated with reduced patient survival rate [43]. Another example is in the regulation of cell turnover in intestinal epithelium where, subsequent to injury, various intestinal cell populations are reprogrammed through mechanisms such as ECM protein-mediated yes-associated protein/transcriptional coactivator with PDZ-binding motif (YAP/TAZ) activation; however, chronic activation and promotion of this plasticity can lead to neoplasia [44]. This overlap in signaling pathways between regeneration and cancer extends to many biophysical processes involving regulation of plasticity, which is discussed in later sections.

## Biophysical controls

**Fig. 2:**
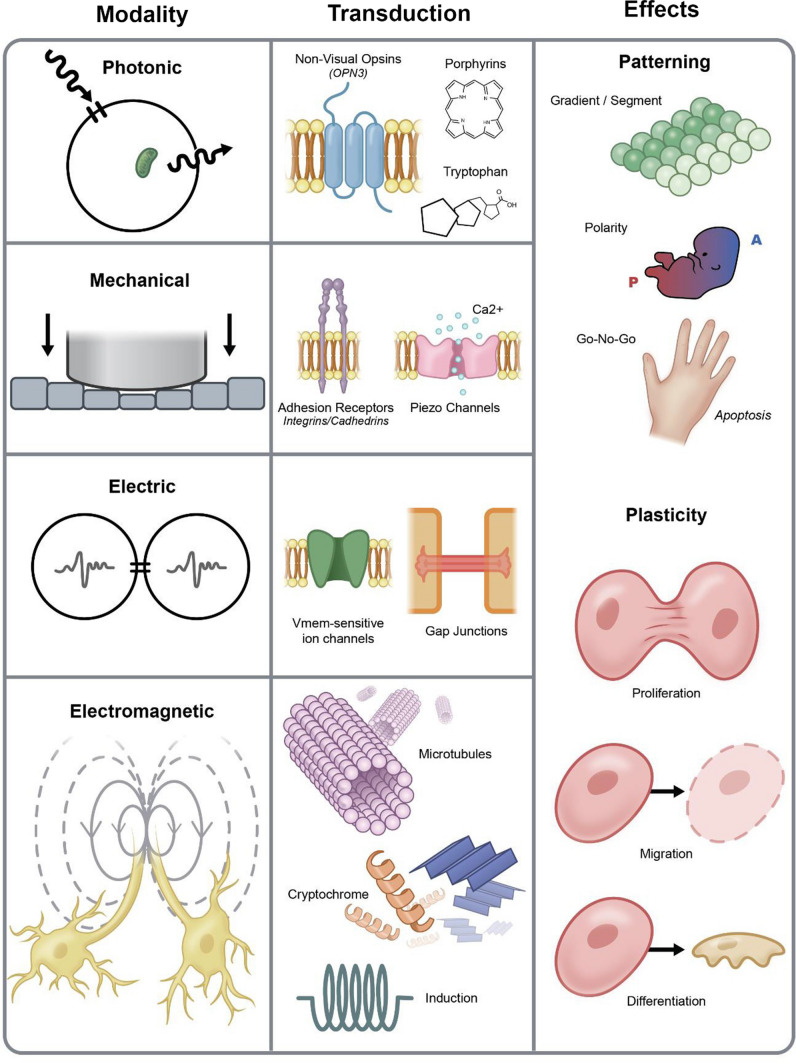
Biophysical modalities, transduction mechanisms, and effects. Photonic, mechanical, electric, and electromagnetic modalities constitute the major biophysical control mechanisms of cellular communication. Each modality is governed by organelles, molecules, and specialized interactions that transduce physical energies into electrochemical signals and their downstream cellular correlates. The effects of biophysical controls on tissue patterning and plasticity include the cell proliferation, migration, and differentiation toward the formation of polar tissues with intrinsic gradients and complex morphologies that contribute to carcinogenesis and regeneration

In addition to biochemical signaling and molecular pathways, there are at least three biophysical modalities that allow cells to share information with each other and their surrounding microenvironments: biomechanical, bioelectrical, and bioelectromagnetic [45–47] (Table 1). These signaling phenomena offer several advantages over their chemical equivalents. First, they often rely on wave propagation through a medium, which increases signaling speed relative to the diffusion or active transport of molecules [48]. Second, biophysical signals are less dependent on proximity or localized interfaces; instead, they can travel longer distances (tissue to tissue), often without specialized conduits [49, 50]. Third, unlike typical ligand–receptor interactions, biophysical signals have spectral properties that increase communicative degrees of freedom [51]. Lastly, biophysical quanta may interact with multiple target structures (Fig. 2) simultaneously rather than sequentially binding and dissociating at receptor sites. To expand on the individual properties of different signals, here, we review each modality as a unique control mechanism underlying plasticity and patterning in regeneration and cancer.

**Table 1:** Overview of biophysical effects on cancer and regeneration

Modality	Subtype	Effect on cancer	Effect on regeneration
Biomechanical controls	ECM composition	Promotion of cancer cell migration and metastasis [70] Promotion of cancer cell survival [74–76] Promotion of cancer cell EMT [78–80]	Control of cell differentiation and identity [66] Induction of plastic state [67, 68]
	ECM stiffness and topography	Induction of EMT [95, 320] Promotion of tumor cell invasion [98] Initiation of neoplasia [100–102]	Modulation of cell adhesion and cytoskeletal tension [82, 83] Regulation of plasticity [10, 37] Control of cell differentiation [85, 88]
Bioelectrical controls	Membrane potential	Promotion of cell proliferation [114, 122] Promotion of cell migration [275]	Modulation of cell differentiation [114, 122]
	Ion channel profiles	Modulation of cell plasticity [20, 115, 135] Modulation of oncogene expression [20, 115] Promotion of cell proliferation and migration [138]	Unique combinations of ion channels embedded within the membranes of distinct cell types significantly impacts cell fate and behavior [128] Cell lineage reprogramming [16]
	Gap junctions	Tumor formation and growth [156, 160–162]	Transient GJ densities and sizes in liver regeneration [155] Upregulates plasticity [157] and patterning [158] Re-establish tissue polarity after an injury [159]
	Acidification	Enhances invasion, propagation, drug resistance, cell survival, and aggression in osteosarcomas [70]	Acidification of liver cells along amputation planes [168] Lysosome acidification after injury in zebrafish [169, 170]
Bioelectromagnetic controls	Electric and magnetic fields	Pro- and anti-carcinogenic properties [218] Nanosecond-pulsed electric fields inhibit tumor growth [220–223] Weak, time-varying magnetic fields synergize with carcinogens to further enhance mammary tumor growth [226]	Initiates, enhances, and accelerates wound healing [209, 210] mT-range, time-varying magnetic fields promote diabetic and skin wound healing [211] Electric fields guide stem cell migration for neural regeneration [212]
	Optical signaling	Wavelength-dependent UPE predicts malignancy [197] Colon cancer autophagy and other cancer modulations are induced by blue LED exposure [251, 253]	Wavelength dependence of UPE emissions for different stages of regeneration [183] UPEs track bone growth and fibroblast differentiation [244] Light-based therapies improve healing, promote angiogenesis [245]

### Biomechanical controls

The ability for a tissue or organ to regenerate is dependent on many factors present in the post-injury microenvironment, including biomechanical properties of the ECM such as stress, strain, shear flow, stiffness, and topographical cues [52]. In mammals, the default response to injury is scarring or fibrosis, with the newly deposited ECM structures presenting a significant barrier to regeneration [53]. Fibrotic tissues increase the stiffness of the microenvironment, which disrupts cellular polarity, inhibits regeneration, and promotes malignancy [54]. Indeed, scars are ideal substrates to initiate tumor growth as stiff ECM induces angiogenesis, promotes hypoxia, and inhibits anti-tumor immunity [54]. Non-mammalian species such as axolotls, however, can mitigate these barriers by upregulating matrix metalloproteinases (MMPs), which remodel the otherwise inhospitable ECM, and by initiating signaling pathways to help direct cells to dedifferentiate, migrate, and replace damaged or lost tissues [55]. Thus, the ECM provides biophysical cues to regulate cellular phenotypes and plasticity in several ways from mechanoreception to integrin signaling, ligand binding, biochemical cues, and more [56–58]. Cells can sense mechanical cues via surface receptors such as integrins that adhere to ECM ligands and transmit signals through cytoskeletal elements, facilitating transduction of biomechanical signals into downstream molecular activation. Once transduced, mechanical signals propagate through cytoskeletal filaments and culminate at the nuclear membrane, resulting in changes in histone methylation and thus changes to chromatin architecture and the epigenetic state of a cell [59].

#### ECM composition and adhesion

ECM composition refers to the molecular network of proteins and proteoglycans that make up the natural scaffold of the tissue microenvironment. Modification of the ECM composition as part of the injury response can either promote or inhibit regeneration. Cells interact with surrounding ECM components through various surface receptors leading to activation of downstream intracellular pathways. Following injury in regeneration-competent species such as zebrafish, mechanical waves across tissues signal the position of wounds [60] and pro-regenerative proteins such as laminin and fibronectin are upregulated whereas collagen IV, a major component of fibrotic scars, is downregulated [61]. This contrasts with non-regenerative species, including humans, that experience scarring following injury, characterized by the differentiation of fibroblasts into ECM-synthesizing myofibroblasts [62]. Modulation of ECM protein composition can both maintain a desired cell phenotype (e.g., chondrocytes cultured on decellularized cartilage ECM) [63], and induce dedifferentiation (e.g., secretion of fibronectin, collagens, and hyaluronic acid by cardiac fibroblasts to promote dedifferentiation of cardiomyocytes after injury in regenerative-competent species) [64]. The ECM composition can also change with age; the composition of the cartilage matrix of the rabbit ear changes over time and corresponds to a loss of morphological plasticity in older animals when compared with the cartilage of immature animals [65].

The presence of excessive collagen and fibrinogen changes the composition of the injury microenvironment which elicits important cellular responses including alterations in plasticity and phenotype. For example, following injury to the liver, manipulating the microenvironment to decrease laminin and total ECM concentration leads to increased hepatocyte differentiation to promote liver regeneration [66]. Although plasticity is often thought of as desirable during regeneration to promote processes such as dedifferentiation, epithelial to mesenchymal transition (EMT), and redifferentiation, increasing plasticity can also lead to loss of regenerative ability. In joints, injury or osteoarthritis results in degradation of cartilage including loss of collagen, aggrecan, and other proteoglycans that in turn modulates the plasticity of chondrocytes, promoting their dedifferentiation into a chronic fibrogenic phenotype [67]. Indeed, cell plasticity can become self-limiting if induced differentiation promotes terminal or otherwise anti-regenerative phenotypes. Interestingly, in Duchenne muscular dystrophy, it has been observed that, through a transforming growth factor (TGF)β-dependent pathway, dystrophic muscle cells increase in plasticity with aging to adopt a multipotent, fibrogenic fate [68]. These cells then contribute to the decline in the ability of muscle to regenerate associated with the disease progression, demonstrating that increased cellular plasticity is not always linked to a pro-regenerative state [68]. Understanding the complex relationship between ECM composition and plasticity of important cell types can help develop targeted strategies for manipulation of the ECM in a way that will promote the desired plasticity (or perhaps suppress unwanted plasticity) to achieve regeneration. However, without effective tissue patterning, plasticity is a morphologically aimless process that may not always benefit the organism.

As with normal cell types, tumor cells interact with and are regulated by the surrounding tissues, cells, and ECM, which are collectively known as the tumor microenvironment (TME) [69]. The physical and biomechanical properties of the TME can alter tumor behavior, and tumor cells in turn alter the TME to promote their survival and cancer progression. Mesenchymal stromal cells can alter the stiffness and molecular composition of the TME by secreting cytokines like vascular epithelial growth factor (VEGF) to stimulate metastasis and migration [70]. This suggests that much like how regenerating cells can modulate their environment to promote growth, tumors respond to factors in the TME and alter their properties to promote cancer progression.

The secondary microenvironment of colonized metastatic sites represents another important feature of cancer that is influenced by biophysical properties. It has long been noted that certain cancers are more likely to metastasize to specific organs and that this may be related to physical bottlenecks like anatomical proximity and circulation patterns [71, 72]. In addition, much like in the primary tumor site, cancer cells shape the biophysical properties of the secondary site into a pro-cancer niche that can promote survival and colonization of metastatic cells and overall cancer progression [73]. Interestingly, some cancer cells can send long-distance signals (tissue level, centimeter scale) to macrophages in the secondary site that will favorably alter the ECM microenvironment to prime it for arrival of invading cancer cells [74].

In contrast to their normal counterparts, cancer cells can abnormally alter the TME to promote their survival and progression [75, 76]. Tumor cells drive cancer-associated fibroblasts (CAFs) to alter the ECM via TGF-β, Notch1, and WNT pathways, and crosstalk between these cells and the ECM generates tumor heterogeneity [77]. Cancer cells themselves can also directly interact with the surrounding ECM, exerting traction forces that have been demonstrated to generate dense collagen bundles between cells in a non-transient fashion, such that the bundles remain even after the traction is gone [78]. Furthermore, the tumor ECM is typically described as more aligned than normal tissue counterparts, where protein polymers display marked group polarity [79]. Through aberrant signaling and mechanical forces in CAFs and the cancer cells themselves, the ECM is remodeled to produce parallel organization of the stromal ECM fibrils [78]. This increase in alignment is believed to play an important role in cancer EMT [80].

#### ECM stiffness, stress, and topography

One major difference between scar tissue and regenerative-competent tissue is ECM stiffness, where scars are defined by fibrotic regions of disorganized, rigid collagen polymers [81]. Increased ECM stiffness following injury is cited as a central factor in driving scar formation over regeneration; for example, artificially increasing the load on mouse skin increases fibrosis through a focal adhesion kinase (FAK)-dependent mechanism [82]. Stiffness of the ECM translates into changes in the cell by modulating cellular adhesion and cytoskeletal tension, which in turn influences mechanosensitive ion channels [83]. Integrin-mediated mechanotransduction of stiffness cues results in phosphorylation of transcriptional regulators YAP and TAZ which translocate to the nucleus in response to increasing stiffness [84].

The stiffness of the ECM can affect the plasticity of many cell types via mechanoreceptor-mediated mechanisms. Regulation of stemness has been linked to changes in actin force [85]. For example, softening the culture substrate in vitro results in reprogramming of various stable cell lines via actin and tension-dependent upregulation of stemness-associated genes like Oct4 and Nanog5. The effect of matrix stiffness on cell plasticity in vitro has been extensively reported (10,37). Mesenchymal stem cells (MSCs) and other cell types can be directed into neurogenic, myogenic, or osteogenic lineages in vitro in the absence of soluble reprogramming factors simply by modulating ECM stiffness [86]. In MSCs, this modulation was found to be reliant on tropomyosin (TPM)1, a mechanosensitive differentiation regulator [87]. Furthermore, MSCs can undergo chromatin remodeling to upregulate pluripotency genes like Oct4, Nanog, and Sox2 in response to low-stiffness media (1.5–15 kPa) [85]. In vitro, mouse vascular smooth muscle cells can modulate their plasticity by switching between a dedifferentiated proliferative state, or a terminally differentiated contractile state depending on the stiffness of the ECM (low or high respectively) in a mechanoreceptor-mediated Rac/Rho-YAP/TAZ mechanism [88], and increased ECM stiffness upregulates RhoA and YAP/TAZ to inhibition expression of pro-regenerative markers in peripheral Schwann cells [89].

Available evidence of stiffness as a modulator of cellular plasticity in vivo is more limited. The African spiny mouse is a valuable model for its unique scar-free dermal regeneration [90]. Interestingly, their skin lacks α-smooth muscle actin, and is reported to be 20 times weaker than that of *Mus musculus* [90]. This reduced stiffness of the ECM is thought to be more permissive to scar-free healing, reminiscent of fetal mouse dermal healing. However, to date it is unknown if this example of low stiffness and scar-free healing is linked to regulation of cellular plasticity. Regeneration of zebrafish fins is partially dependent on viscous sheer stress and the resulting internal tension of the fin, which provides signals for guiding fin regeneration after amputation [91]. These natural examples of the control of regeneration through ECM stiffness and other biomechanical cues offer clues as to how we might bioengineer the tissue microenvironments of non-regenerative species to promote regeneration.

In tumors, the TME possesses elevated interstitial fluid pressure (IFP) due to the presence of leaky vasculature and deposition of excess ECM proteins causing local retention of fluid [92, 93]. This increased IFP leads to elevated shear stress acting on TME cells to drive a multitude of plasticity-related downstream changes, including induction of EMT. Research using several cancer cell lines has demonstrated that EMT is elevated in response to elevated shear stress [94, 95] with evidence in MCF7 breast cancer cells that it can also promote a cancer stem cell (CSC)-like phenotype [96]. Shear stress is not only a factor in the TME but also during cancer metastasis. A study by Cognart et al. [97] modeled the effect of shear stress on breast CSCs as they circulated in the vasculature during metastasis, finding that the inclusion of circulation induced significant changes in gene expression, particularly EMT markers [97].

Osteosarcoma cells have been observed to respond to ECM stiffness via an integrin-mediated FAK signaling pathway [63, 70], which promotes migration/invasion and angiogenesis [98]. In addition, high-stiffness ECM can drive the EMT, cell invasion, and metastasis in breast cancer cells by promoting ligand-independent phosphorylation of EphA2, which recruits and activates Lyn kinase, resulting in phosphorylation of Twist1, a pro-EMT protein [99].

In one of the most well-studied examples of the role of matrix biophysical properties in cancer, ECM stiffness has been found to play a role in the onset of neoplasia. Cancers can arise when cells lose their ability to sense the rigidity of their local ECM via interference with mechanoreception pathways, such that cancer cells become incapable of responding to mechanosensory growth inhibition [100]. When expression of cytoskeletal components such as tropomyosin is induced in cancer cells, rigidity sensing is restored and growth is inhibited [101]. Conversely, inhibiting other components of the rigidity-sensing complex promotes transformed growth of cells into neoplasia [102]. Understanding the cues that trigger this change in phenotype and transformation to a more plastic state may contribute to the development of therapeutics that can target these pathways to inhibit neoplasia at an early stage.

### Bioelectric controls

Cell membranes are semi-permeable boundaries across which physiological ions are transported to generate chemical energy [103], activate secondary messengers [104], initiate transcription [105], mobilize cytoskeletal changes [106], induce mitosis [107], as well as conduct local and long-range signaling [108]. While membrane potential (*V*_mem_) is most often associated with excitable cells such as neurons in the brain and cardiomyocytes in the heart, electrical signaling is a generalized feature of most cells [109]. Indeed, cellular respiration by mitochondria is effectively a bioelectric phenomenon as the canonical reactions are contingent upon the activities of ion channels and pumps [103, 110]. Oogenesis, cell growth, and proliferation are all associated with sharp influxes of calcium ions [111], which, in addition to their myriad chemical interactions, carry considerable positive charge and the ability to rapidly depolarize membranes, which are typically hyperpolarized at rest. Because calcium is also a well-known second messenger that promotes the expression of morphogens (e.g., Nodal, sonic hedgehog (SHH), bone morphogenic protein (BMP), TGF-β) [112], and disruptions of bioelectric networks have been linked to impaired body planning, it is worth considering the role of bioelectricity in tissue plasticity and patterning in the context of regeneration [113]. Similarly, the bioelectric states of cancer cells and the tumors they form differ markedly from healthy, somatic counterparts [114]; however, they share significant overlap with those of embryonic and regenerating tissues. Hallmarks include significantly depolarized *V*_mem_, electrical isolation from local cell populations, and aberrant ion channel profiles [115], which can drive gene expression, inhibit apoptosis, and elevate a dysregulated plasticity, favoring a tumorigenic state.

#### Membrane potential

The functional role of *V*_mem_ has been a subject of intense debate in several subfields of biology. One major area of contention is the classification of *V*_mem_ as either an active participant in cell and tissue dynamics or as an epiphenomenon with little physiological relevance beyond representing a reporter of cell states. Examining the range of resting potentials associated with most cell types (− 10 to − 90 mV), it is clear that *V*_mem_ predicts proliferation and differentiation potential [114]. For example, somatic cells are generally hyperpolarized at rest (− 50 to − 90 mV) with transient depolarizations and re-polarization events. Among somatic cells, the most non-proliferative and terminally differentiated cells display the greatest membrane polarity, such as cardiomyocytes (− 90 mV) and neurons (− 70 mV)—these tissues are also the most resistant to regeneration and are often post-mitotic [114]. However, highly regenerative liver cells and stem cells as well as cancer cells generally display depolarized *V*_mem_ at rest (0 to − 50 mV) [114]. During malignant transformation and proliferation, *V*_mem_ depolarizes significantly [116]. On the bases of these observations alone, it is difficult to determine the possibility of cause-and-effect relationships. However, systematic manipulations of *V*_mem_ have been used to biophysically control cell function. Indeed, mitosis can be inhibited by altering the extracellular medium and forcibly hyperpolarizing cells [117]. Interestingly, otherwise terminally differentiated mature neurons can be induced to divide under chronic depolarizing conditions [118]. While neurons regularly undergo acute depolarization events over a few milliseconds termed “action potentials” (which are also accompanied by mechanical waves) [119], only chronic depolarization generates the mitotic phenotype. Stem cells are also known to alter their secretions, shapes, and migration trajectories when activated by bioelectric signals [117, 120, 121]. Notably, the types of channels that drive *V*_mem_ fluctuations are relevant to biological outcomes due to their unique association with specific ionic currents as well as their densities and intrinsic time constants. A cell’s complex “ion channel profile” is, therefore, critical to its function.

The resting *V*_mem_ of proliferative cells, including cancer cells, is significantly depolarized relative to differentiated cells [114]. Quantitatively, the discriminant threshold between these two populations is approximately − 36 mV [122]; however, cancer cells display even greater depolarizations than their non-cancerous, proliferative counterparts [114, 122]. This is consistent with the observations that stem cell differentiation is dependent on hyperpolarization and inhibited by depolarization [123]. As was described in a comprehensive review by Yang and Brackenbury [116], depolarized resting *V*_mem_ in cancer is maintained by ion channel profiles that elevate, among other parameters, intracellular Na^+^ rather than K^+^, ultimately favoring cell proliferation and migration over differentiation. Interestingly, facilitating anion transport in cancer stem cells hyperpolarizes *V*_mem_, triggers differentiation, and promotes cell death [124]. However, *V*_mem_ is not static in cancer cells and transient hyperpolarization events may be necessary to initiate cell cycle progression [125, 126]. Finally, because tumors are characterized by disorganized cytoarchitectures that often lack polarity, it is likely that meso-scale bioelectric networks are significantly disrupted in addition to abnormalities within individual cells. Dynamic, multi-scale models of bioelectricity are needed to parse these contributions, inform experimentation, and guide clinical applications.

#### Ion channel profiles

Ion channels define the bioelectric states of cells. Several stimuli and environmental conditions can trigger ionic current flow including electrochemical gradients (e.g., potassium leak channels), *V*_mem_ fluctuations (e.g., voltage-gated calcium channels), molecular ligands [e.g., ionotropic channels such as N-methyl-D-aspartate receptors (NMDARs)], biomechanical signals (e.g., piezo channels), proton concentrations (e.g., acid-sensing ion channels or ASICs), photons (e.g., opsins), and other energy sources [127]. Variation of ion channel profiles—the unique combinations of ion channels embedded within the membranes of distinct cell types—can significantly impact cell fate and behavior [128]. For example, in response to experimentally lesioning rat brains, astrocytes lacking inward-rectifying potassium (K^+^ Kir) channels were induced to proliferate, forming scar tissues that impeded regenerative potential [129]; however, astrocytes that could re-polarize their membranes did not respond similarly. Indeed, ion channel profiles may play a critical role in lineage reprogramming [130].

Several ion channel-dependent mechanisms of proliferation, migration, and suppressed differentiation in cancer cells have been described in the literature [131, 132]. Blocking several types of Na^+^, K^+^, and Cl^−^ channels can modulate the cancer state [20, 115]. In many cases, calcium (Ca^2+^) influx represents a convergent point for signaling [133]. Inward-rectifying (e.g., Kir) and depolarizing channels represent an important dimension of this dynamic [134]. As *V*_mem_ shifts toward a depolarized state, the resting potential approaches the activation threshold for voltage-gated calcium channels, which can trigger cell migration, cell cycle progression, and proliferation. Paradoxically, hyperpolarizing channels can generate favorable electrochemical gradients for Ca^2+^ influx with the same net effect as direct depolarization. However, forced expression of hyperpolarizing ion channels can significantly reduce oncogene-induced tumorigenesis [20, 115]. Overexpression of Kir 4.1 channels in gliomas, which are normally absent in tumor cells of glial origin, hyperpolarize *V*_mem_, suppress growth, and promote differentiation into somatic cells [135]. Genes regulating differentiation are responsive to *V*_mem_ fluctuations and depolarization may serve to maintain cells in an undifferentiated state [136], which can be ideal as a transient response to injury in the case of regeneration but highly disruptive within normal tissues as is the case with cancer.

At the intersection of biomechanics and bioelectrics, piezo channels (piezo1 and piezo2) are cation-permeable mechanotransducers that are expressed in many different tissues from sensory neurons to kidney mesangial cells, and skeletal myotubes, where they regulate differentiation, migration, and proliferation [137]. Responding to shear flow, stretching, stiffness, topology, compression, and osmotic stress, piezo channels are an important part of the overall ion channel profile, where they transduce mechanical forces within the cytoskeleton or throughout the cell membrane to impact the bioelectric dynamics of regenerative and cancerous tissues. Piezo channels are overexpressed in several cancers, where influxes of calcium drive tumor progression [138], glioma aggression [139], and metastasis [140]. Ca^2+^-permeable piezo channels are known to inhibit axonal regeneration in Drosophila [141]; however, they have been implicated in neural stem cell differentiation by way of myosin II activation [142]. While piezo channels are considered an important interface with which to harness ECM stiffness modulation for improved cancer therapy [143], there are likely many unexplored applications. Indeed, the emergence of novel mechanostimulus-driven cancer therapeutics and functionalized materials to locally modulate shear forces, compression, and tension within the tissue microenvironment may indicate a trend in this direction [144].

#### Gap junctions

Gap junctions (GJs) are specialized channels that connect the intracellular spaces of adjacent cells, allowing the passage of ions as well as small molecules (< 1.5 kDa) including inositol trisphosphate (IP3), glucose, ATP, peptides, siRNAs, and amino acids [145]. GJs are important players in bioelectric networks because all physiological ions can pass through GJs. As cells coupled by GJs share a continuous plasma membrane, they display highly responsive electrotonic signaling capacities. Thus, large tissue areas can be synchronized by GJs, sharing *V*_mem_ to generate meso-scale and long-range tissue polarity associated with left–right patterning and the establishment of body axes [146, 147]. Normal developmental processes including embryogenesis and the activities of stem cells are critically dependent on GJ-related events such as calcium waves [148, 149]. For example, tissue patterning dynamics including the directional outgrowth of feathers in developing chicks, as well as the precise spacing between their limb buds, are determined by Ca^2+^ and GJ-mediated depolarizing waves that trigger mesenchymal stem cells to differentiate [150]. Indeed, factors associated with pluripotency (e.g., nanog, sox2, Oct4) are known to activate the expression of GJs to maintain embryonic stem cell phenotypes [151]. Further, morphogens such as BMP are active participants as modulators of GJ intercellular communication [152, 153]. Consistent with the role of GPs as mediators of plasticity and patterning, blocking or altering GJ function can cause teratogenic injuries, characterized by highly disorganized tissue patterning [154].

As regeneration is fundamentally a transient recapitulation of developmental pathways, it is unsurprising that blocking electronic signaling can also impact the ability for tissues to re-pattern themselves after an injury. Liver regeneration is marked by transient changes in GJ densities and sizes within cell membranes [155]. When GJs become dysfunctional in the highly regenerative liver, hepatic cancer becomes more likely to develop [156]. Even organs with low regenerative potential are impacted by GJ function. When bone-marrow-derived progenitor cells (BMPCs) are injected into damaged heart tissues for myocardium regeneration, the establishment of GJs between resident cardiomyocytes and BMPCs promotes plasticity, triggering differentiation along the cardiogenic fate lineage [157]. Patterning of regenerated tissues is also affected by GJs. Indeed, bisected flatworms display atypical morphologies including multiple head regions upon regeneration when GJ communication is blocked or subject to loss-of-function manipulations [158]. One proposed mechanism, which is consistent with the hypothesis that bioelectricity is a key determinant of regeneration, suggests that GJs work to re-establish and stabilize tissue polarity after an injury [159]. That is, without a capacity to rapidly synchronize the bioelectric networks of tissues, re-patterning fails due to a breakdown of biophysical communication among cells.

Gap junctions are also important regulators of cell fate and behavior in cancer. When GJs are blocked or downregulated in animal models, tumors spontaneously form as evidenced by pharmacological assays and knockout studies with mice [160, 161]. In the absence of GJs, as cells become electrically isolated from each other, the likelihood of tumor formation increases markedly [74]. This is consistent with the observation that many types of tumor cells are deficient in connexin expression [162]. Furthermore, transfection of cancer cells with connexins restores GJ signaling and suppresses tumor growth [163]. Interestingly, the relationship between cancer and GJs may be reciprocal as oncogenes such as Src can regulate GJ communication, providing a potential mechanism for positive feedback loops of increased bioelectric dysregulation [164, 165].

#### Acidification

Bioelectric signaling arises from disparities of charge across membranes, often driving ionic currents along electrochemical gradients or gating specialized channels that respond to *V*_mem_. Notably, ion channels can respond either directly (e.g., ASICs) or indirectly to local pH changes in the extracellular environment [166]. Thus, chemical changes within the regenerative microenvironment may feedback into biophysical modulators as the extracellular concentration of positively charged hydrogen ions (H^+^) increases [167]. Indeed, acidification has recently been identified as a key factor in early stages of the regeneration process. Mammalian livers are capable of excellent regeneration, and rat models have demonstrated that post-hepatectomy there exists a transient acidification (with peaks around 3 h post-amputation) of hepatocytes proximal to the amputation plane, which promotes ectopic ATP synthase activity in these cells [168]. Acidification of lysosomes post-injury has also been observed in zebrafish fins, and inhibition of this process, which can be achieved by systemic glucocorticoid treatment, prevents the activation of downstream regenerative processes such as growth factor expression and blastema formation [169, 170]. Although the mechanistic details by which this process occurs have not yet been fully elucidated, it highlights that this sudden yet transient drop in pH plays an important role in the regenerative process. Perhaps the most direct link between pH as a modulator of bioelectric states in regeneration is the involvement of proton flux. For example, V-ATPase H^+^ pumps are upregulated at the cell surface in amputated Xenopus tails, where they drive the efflux of protons at the wound edge, generating increased polarity across the tissue relative to a distal depolarized region [171]. Other known contributors to regeneration, including potassium and calcium ion channels, are modulated by the electrochemical gradients formed by local pH changes [107]. In cancer, acidification of the TME has been shown to enhance invasion, propagation, and drug resistance, as well as cell survival and aggression in osteosarcomas, operating via a calf intestinal alkaline phosphatase (cIAP)/TNF receptor-associated factor (TRAF)/ nuclear factor (NF)-κB pathway [70]. Because H^+^ is a well-known activator of TRP channels [172], which are generally permeable to cations and thus contribute to depolarization, local acidification of tissues should be considered as a potential modulator of cell states including in cancer.

### Bioelectromagnetic controls

Electromagnetic (EM) signals are receiving increased attention as relevant biophysical modulators of tissue plasticity and patterning [173]. Cells shuttle ions across their membranes, generating current with associated electric and magnetic fields. Unlike molecular signals, EM signals are not constrained by chemical bottlenecks such as diffusion, enzymatic degradation, and local concentration gradients. Cellular EM fields are detected by neighboring cells and are known modulators of bioelectric signaling [174]. EM fields can re-orient the cytoskeleton and selectively activate ion channels [175, 176]. Perhaps most relevant to regeneration are the well-characterized galvanotactic gradients that direct cell migration within tissues [176]. Their relevance is evidenced by the contemporary use of applied electric gradients as guides for oriented tissue regeneration and growth [177–179]. Further, biomedical techniques that employ more intense EM fields such as electropermeabilization extend the utility of EM as a tool for targeted tissue re-patterning [180, 181]. Beyond EM fields, biologically generated photon emissions (termed ultraweak photon emissions (UPEs) or “biophotons”) have been linked to mitogenic processes such as wound healing and optical signals such as pulsed light have attracted attention as potential mediators of regeneration [182, 183]—even in classically anti-regenerative tissues such as the brain [184].

Can electromagnetic factors influence cancer? There is a well-known, long-standing association between childhood leukemia and EM field exposure from high voltage powerlines [185–187]. There is also evidence that indicates EM radiation from cellphones may contribute to increased incidence of brain cancer and other pathologies [188, 189]. Considering EM fields affect cell migration, proliferation, and gene expression in vitro [190–192], it would be unsurprising to discover a defined mechanisms relating EM fields, tissue plasticity, and patterning that could drive or inhibit carcinogenesis. However, it is likely that EM field pattern (frequency modulation) and intensity (amplitude modulation) are critically important determinants of outcomes because many sources of EM radiation are not carcinogenic [193, 194]. Indeed, EM-based therapies have been developed which involve applications of electric or magnetic fields, direct electric current, and even pulsed light to treat cancer [195, 196]. Cancers, of all sub-types, emit UPEs with fingerprint-like spectral profiles (wavelength) and release patterns that are distinct from healthy tissues [197, 198]. Thus, light may be used as a biomarker of disease states including as a diagnostic marker of cancer. Importantly, EM factors do not represent an intrinsic hazard or toxin—nor do they represent a panacea. Rather, the preponderance of evidence suggests that EM factors influence fundamental biological processes that either directly or indirectly affect cell fate and behavior. Therefore, as our understanding of EM signaling increases, it becomes increasingly likely that many biomedical applications will soon follow.

#### Electric and magnetic fields

Electric and magnetic fields are distinct components of a shared EM modality that can interact with biological systems. While static charges such as meso-scale tissue polarity can generate electric fields, moving charges associated with ionic currents at the micro-scale are needed to generate magnetic fields. Thus, different bio-EM interactions are expected at different scales. Endogenous EM field emissions can be detected at the surface of the skin, tracking changes associated with growth, maturation, and regeneration of tissues [175, 199, 200]. Skin wounds in mammals generate endogenous electric field responses lateral to the wound center with intensities of ~ 150 mV/mm [201, 202]. Very weak currents (µA/cm^2^) can even be detected leading from the wound edge to its center, with the potential to guide cell populations to and from the injury site [203]. Cell migration along electric fields (galvanotaxis) is a well-documented phenomenon, where the direction is typically toward the positively charged cathode; however, some cell types including macrophages can be induced to migrate toward the anode [204]. Directional migration is possible because the intracellular space holds an intrinsically negative charge due to the presence of anionic molecules such as DNA, RNA, and phosphorylated proteins, and this bias toward polarized resting states is further reinforced by hyperpolarizing ion channels. However, the cell can also be considered an independent polar object, with inherent asymmetries (e.g., microtubule-organizing center location, ion channel distribution, nucleus location, etc.) that may contribute to its local EM-based response patterns. Indeed, because free-floating microtubules spontaneously align with electric fields in vitro [205], their role as endogenous EM biosensors must be considered seriously. As cells form tissues with specific orientations, endogenous EM field complexity likely increases, contributing to multiform EM landscape within the body. There is now overwhelming evidence that endogenous EM fields orchestrate brain function including coherent oscillations and memory-forming functions associated with synaptic plasticity [206–208].

Applied magnetic fields and electric fields have been used to initiate, enhance, and accelerate wound healing [209, 210]. In rats, low-milliTesla (mT)-range, time-varying magnetic fields have been used to heal cutaneous wounds while diabetic wounds have been treated with higher intensity (180 mT) static magnetic fields [211]. Electric fields have been used to guide stem cell migration for neural regeneration and nerve regrowth can be enhanced with weak currents (10 µA/cm^2^) and field strengths approximating 100 mV/cm [212]. Interestingly, low-frequency (< 100 Hz) EM field applications preferentially differentiated neural stem cells into astrocytes or neurons [213], suggesting a potential frequency-dependent role of EM fields in lineage programming. There is also evidence to suggest that direct applications of electric current can help regenerate bone and even spinal cord tissues [214, 215]. Electroacupuncture has even been used to stimulate peripheral nerve regeneration [216, 217]. Because life on Earth is constantly immersed within both natural and artificial EM fields including the geomagnetic field as well as those from high voltage power lines and other electronic circuits, it is worth considering how environmental influences may affect wound healing and regeneration.

EM fields and applied currents are known modulators of cancer, with both pro- and anti-carcinogenic properties that are critically dependent on the frequency and amplitude of the applied signal. Interestingly, endogenous EM fields have been observed in malignant tissues [218]. The direction and magnitude of EM fields across tumors are highly variable due to the disorganized nature of the cytoarchitecture [219]. Because different cell types display unique responses to different EM field thresholds, effects of applied fields are expected to be highly context-dependent. High-intensity (300 kV/cm) nanosecond-pulsed electric fields have been used to inhibit tumor growth, likely by apoptosis [220, 221]. However, low-intensity (1–2 V/cm) applications have also proved effective at similar and other frequencies, particularly as a targeted treatment of glioblastoma [222, 223]. The tumor-promoting effects of ambient magnetic field conditions associated with commercial electrical systems and lighting, with alternating frequencies within the 50–60 Hz are not clear as mixed results suggest unidentified complex interactions are likely [224]. Magnetic fields may promote or co-promote tumor growth under certain conditions [225]. Indeed, very weak (100 µT), time-varying magnetic fields enhanced mammary tumor growth in female rats exposed to a chemical carcinogen (7,12-dimethylbenz(a)anthracene or DMBA) [226]. Cancer-related gene expression was found to increase in cells exposed to low-mT magnetic fields for 24 h [227, 228]. Static magnetic fields can stimulate the secretion of ECM components, trigger specific types of calcium receptors, and modulate cytokine release [229, 230]. Alternating and/or pulsed magnetic fields induce current across tissues with minimal heating below 100 kHz. In addition, interactions between EM signals and other components of the tissue microenvironment can be quite complex. Galvanotactic experiments with brain tumor-initiating cells demonstrate that, upon exposure to direct current electric fields, migration toward the anode or cathode is dependent upon the substrate of the ECM (e.g., laminin, collagen, hyaluronan) and whether it was 2D (monolayer) or 3D (hydrogel)—a biophysical interaction between EM and mechanical factors involving topology [231]. Therefore, applied fields for therapeutic interventions are expected to interact with the tissue microenvironment.

#### Optical signaling

Spontaneous UPEs, with intensities approximating 10^−15^ W/cm^2^ and wavelengths ranging from 200 to 1300 nm, have been measured as endogenous EM-related signals from cells and tissues [232, 233]. Their fluctuations are linked to cell cycle progression, microtubule dynamics, cellular respiration, neurotransmission, and *V*_mem_ fluctuations among other correlates [234]. UPEs are generated by several biochemical mechanisms including lipid, nucleic acid, and protein oxidation reactions, mediated by reactive oxygen (ROS) and nitrogen species (RNS) [235, 236]. Thus, light emissions are readily observed in stressed, aging, diseased, or highly metabolic systems [237, 238]. As excited electrons return to ground state, photons are released with emission frequencies and patterns reflective of molecular events within the cell [239]. Though optical signaling among cells has not received significant attention, the widespread expression of non-visual opsins (e.g., OPN3, OPN5) in tissues of the eyes, skin, brain, testis, spinal cord, lung, liver, and kidney among others [240] suggest that they may serve a physiological role. In fact, light-based communication among neurons is an active area of investigation and inspiring the development of novel technologies to non-invasively measure biophysical states [241–243].

Optical signaling is perhaps the most understudied biophysical modality in the field of regenerative biology. However, UPEs have been investigated as correlates of wound healing in animals. In one study, light emissions with wavelengths between 230 and 700 nm were detected with unique signatures associated with inflammation and proliferation phases in rodent models [183]. Light emissions have also been used to track bone growth and fibroblast differentiation [244]. Interestingly, artificial sunlight irradiation of skin fibroblasts induces ultraweak photon emission and may even be transiently stored by cells [245]. Light therapies, involving wavelengths within the infrared and visual spectra, have been used in recent years to improve healing in retinal, vascular, and dermal tissues, and wavelength-specific light exposures may even induce patterned angiogenesis [245]. There is a current need to explore the role of UPEs in regeneration as well as potential light-based therapies and their underlying mechanisms.

Endogenous photon emissions are coupled to many classic cancer biomarkers. UPEs correlate with stress indicators such as ROS and RNS, microtubule dynamics, cell cycle progression, and gene transcription [235, 236]. Notably, wavelength-dependent signatures of UPE emissions (using filters for 420 nm, 620 nm, and 950 nm), including the ratio of infrared-to-ultraviolet emission counts, can discriminate malignant and non-malignant cancer cells and are predicted to converge with the spectral properties of delocalized electrons from linearized peptide chains in proteins associated with gene expression [246]. It may even be possible to discriminate cancerous and non-cancerous cells using very narrow-band filters for UPEs around 500 nm [247]. Further, when melanoma cells die, the spectral characteristics of their UPEs shift markedly and respond with specificity to different biochemical activators and blockers [248]. Mechanical factors may also influence biophoton emissions. Indeed, photon emission from tumor cells can be enhanced by 3 MHz ultrasonic stimulation (mechanical vibration) with distinct changes relative to healthy liver and spleen tissue [249]. Like many other cells, cancer cells express non-visual photoreceptors such as Opn3, which exhibits optimal absorption around 460–470 nm—values that are well within the range of endogenous UPE emission spectra [240]. Therefore, endogenous optical signaling in cancer is a relevant phenomenon that should be further explored.

The use of applied light as an external control of cell states has become increasingly popular. While many approaches incorporate the use of nanoparticles, porphyrins, and other photoactive molecules that act as guides for therapeutic exposures of light [250], simple exposure to light itself may be sufficient to modulate cancer [251]. A systematic analysis of laser light irradiation (intensity of 20 J/cm^2^) with multiple wavelengths (*λ* = 410 nm, 488 nm, 630 nm, 635 nm, 640 nm, 805 nm, and 1,064 nm) demonstrated that the mitotic rate of tumor and normal cells could be modulated by wavelength-specific light [252]; however, similar techniques with lower intensity had different biomodulative effects. Using blue LEDs (465 nm), which converge with the spectral characteristics of Opn3, investigators have demonstrated the ability to induce autophagy in colon cancer cells [253]. Similar conditions are associated with the inhibition of colon cancer and the promotion of cancer-associated fibroblasts [254], which can drive tissue microenvironment reorganization associated with tumors.

## Engineered applications of biophysical control

**Fig. 3:**
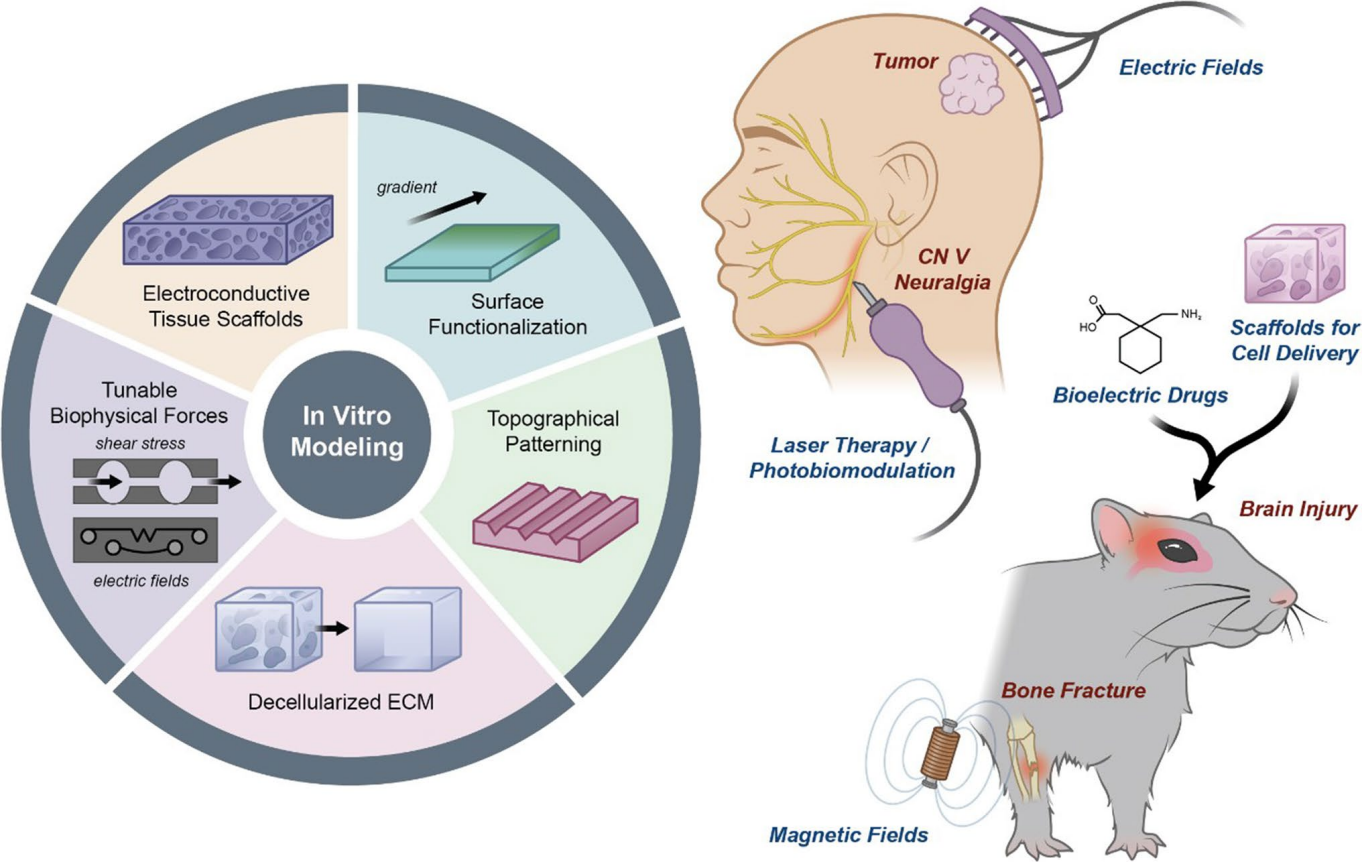
Bioengineered applications of biophysical controls. (Left) In vitro models can be constructed and tested by tuning properties that simulate the biophysics of tissue microenvironments. (Right) Diseases and injuries (red) can be mitigated, inhibited, or reversed by applications of electric fields, light, ECM-simulating scaffolds, compounds that modulate bioelectricity, or by magnetic field exposures

As the biophysical dimensions of cellular communication and their roles as regulators of tissue dynamics have gained recognition, biomedical engineers have harnessed the underlying principles to design novel techniques to reprogram, re-pattern, and replace tissues (Fig. 3). Bioengineered in vitro models allow for the isolation and examination of discreet biophysical mechanisms in well-defined materials that mimic physiological environments. As more devices that allow for precise control of these important biophysical properties in vitro are developed, we can begin to identify and isolate cues to translate their use into in vivo models to further understand plasticity and patterning as products of cellular communication.

### *Surface* functionalization *of materials*

The development of surface functionalization techniques allows an unprecedented control over biomaterial properties such as ECM ligand type, density, or patterning, and stiffness [255]. For example, Smith et al. [256] achieved direct reprogramming of mouse fibroblasts into cardiomyocyte-like cells using an engineered poly(ethylene glycol) (PEG) hydrogel presenting various concentrations and density of laminin and RDG peptide [256]. One particularly compelling use of surface functionalization to study and manipulate cellular plasticity is the creation of patterns and gradients using custom hydrogels and microfluidic devices. During embryo development, there are many important ECM protein and stiffness gradients that drive morphogenesis. For example, in the embryonic mouse limb bud, an ECM stiffness gradient matched by fibronectin expression directs MSC migration during limb development (i.e., durotaxis) [257]. Indeed, much of the work investigating the effect of biophysical gradients has focused on cell migration, but this may also prove to be an important factor to incorporate into developing strategies to promote regeneration through modulation of plasticity.

### Topographical patterning

Topographical patterning is also an important bioengineering strategy to modulate cell fate and plasticity. Using various techniques [258], nano- and micro-topographical cues have been developed for *in* vitro systems that mimic the topography of natural tissue to study its effect on cells, or to promote a certain phenotype or reprogramming of cells. For example, fabrication of parallel microgrooves and nanofibers can direct cell morphology and enhance reprogramming efficiency of mouse fibroblasts through mechano-modulation of the epigenetic state [259]. Many gastrointestinal and colorectal cancers are difficult to model in vitro, as the in vivo structures are tubular, which is not well-represented by 2D epithelial culture. However, representative intestinal organoids can be generated using lipid bilayer-supported droplet networks to form 3D tubular structures [260, 261]. Similarly, to recapitulate the unique structure of cortical folding associated with developing brain tissues, 4D bioprinting techniques have been combined with cell-infused “smart” materials that can be activated to change their shapes by pulses of infrared light [262]. Furthermore, etched surfaces and microfluidic conduits have been developed to guide both neurites and vascular structures to generate polarized tissue structures that can more precisely mimic their natural templates [263]. The continued development of these kinds of 3D culture systems will provide accurate in vitro models that could mimic the ECM or cell–cell interactions present in vivo.

### Tunable flow and shear stress

Bioengineered platforms can also be used to study the biophysical cues that influence cellular plasticity by incorporating properties such as fluid shear stress, ECM stiffness, and electric fields. Controlling ECM stiffness in vitro is accomplished in many ways, such as tuning the substrate concentrations [e.g., of polydimethylsiloxane (PDMS)], or chemical or UV crosslinking [264]. To mimic biophysical phenomena, cells may experience in vivo such as flow-induced shear stress, various in vitro devices have been developed to artificially introduce shear force into a system. Van Haaften et al. [264] created a physiologically relevant 3D model of hemodynamic loading to study the contribution of shear stress and cyclic stretch on tissue regeneration in vascular grafts. They reported that when human macrophages and myofibroblasts were cultured in this bioreactor, the effects of shear stress included modulation of myofibroblast phenotype [265]. Low interstitial flow has also been investigated for wound healing applications [266], for example, to induce fibroblast to myofibroblast differentiation in an α1β1 integrin-dependent process in vitro [267]. Fluid shear stress has also been studied in cancer using innovative bioengineering techniques, including the integration of perfusion and flow within microfluidic and bioreactor platforms [97, 268]. More work is needed to determine how the manipulation of shear stress in vitro may translate to strategies to induce tissue regeneration.

### Decellularized ECM

The use of decellularized ECM from the organ or tissue is an alternative means by which in vivo biophysical properties such as 3D architecture, stiffness or composition can be retained. Once decellularized, this ECM can then be digested and resuspended into a hydrogel or seeded directly with the cells of interest and maintained in culture to study the effects of the ECM properties on cell phenotype and plasticity. Decellularized ECM can also be used to drive the differentiation of seeded cells for transplantation back into the body. One of the most commonly used reconstituted ECM substrates is Matrigel, which is naturally secreted by Engelbreth-Holm-Swarm mouse tumor cells. Interestingly, at microscopic scales, this material displays marked mechanical heterogeneity with punctate regions of increased stiffness relative to the bulk [269], consistent with the natural tumor microenvironment [270]. Hydrogels constructed with varying degrees of Matrigel density were shown to control stiffness-dependent YAP localization in human pluripotent stem cells with downstream effects on cell fate [271]. Other hydrogels, fabricated from decellularized ECM from various cardiovascular tissues and seeded with adipose stromal cells (ASCs), influence cell differentiation differently, likely due to differences in ECM composition of the tissues [272]. Similarly, scaffolds composed of decellularized meningeal tissue were shown to promote the adherence, differentiation, and viability of neural precursor cells [273].

### Modulation of bioelectric activity

Investigators are now exploring the possibility of inhibiting tumor growth by targeting ion channels. Blockades of serotonin-gated Na^+^/K^+^ channels hyperpolarize colorectal cancer cells in vitro with downstream proapoptotic effects and cell cycle arrest [274]. Similar effects have been achieved using antiepileptic drugs that block voltage-gated sodium channels [275]. Regeneration may also be affected by transient sodium currents and mediated by voltage-gated channels as was demonstrated by tail regrowth and patterning experiments in a Xenopus model [276]. As drug delivery methods continue to advance, including the development of wearables with timed release of drugs at the wound interface [277], the possibility of restoring form and function with bioelectric modulators becomes increasingly likely.

While voltage-gated calcium channels can be markedly underexpressed across several types of cancer including brain, lung, kidney, and breast [278], it was recently demonstrated that voltage-gated calcium channel (VGCC) blockades can inhibit tumor growth with synergistic effects when combined with chemotherapies [279]. Interestingly, some authors have reported increased risk factors of cancer in hypertensive patients using VGCCs [280], indicating a more complex relationship requiring further study.

Localized electrical stimulation of the wound site by implanted EM field generators—also known as electroceuticals—is an emerging technology with promising effects on regeneration. Building on seminal experiments in the 1970s, platinum and silver-wired electrodes coupled to embedded resistors were recently implanted within the stumps of amputated rat limbs to deliver low-voltage DC stimulation to enhance regeneration [281]. The stimulation-based treatment induced significant regrowth of bone, cartilage, and vasculature while suppressing the formation of neuromas—indicating significant re-patterning potential. Several studies have demonstrated similarly promising effects on peripheral nerve regeneration with growth-associated gene expression [282, 283]. Earlier works also demonstrated that direct current (0.6 mA), applied for 15 min per day over a period of 9 consecutive days, could be used to reliably generate necrotic lesions within tumors [284]. Similarly brief, low-intensity currents delivered to cancerous tissues were demonstrated to be safe with regard to the surrounding healthy tissues [285]. Recent efforts have demonstrated that vascular normalization, which involves a suppression of pathological angiogenesis and promotes the effects of anti-cancer drugs, can be achieved by wireless electrical stimulation of tumors using polarized ferroelectric nanoparticles; effects which were attributed to disruptions of intracellular Ca^2+^ gradients and endothelial nitric oxide synthase expression [286].

Materials can also be used to focus or amplify endogenous bioelectric signaling for tissue engineering purposes. Indeed, recognizing the role of the microenvironment on electrical signal propagation, neural stem cell differentiation was modulated within custom scaffolds by increasing the electrical conductivity of the substrate (poly(3,4-ethylenedioxythiophene), polystyrene sulfonate or “PEDOT:PSS”) to build models of the central nervous system in vitro [287].

### Applied electric and magnetic fields

To model electrotaxis in vitro, there is a growing number of researchers developing devices to generate electric fields to modulate tissue dynamics [288]. While it is well established that electric fields can drive migration of many different cell types [289, 290], the latest data indicate that EM field stimulation at low (30–300 kHz) and extremely low (3–30 Hz) frequencies can modulate the fate and behavior of stem cells including effects on proliferation, differentiation, cell cycle progression, viability, morphology, and metabolism [291]. It was recently demonstrated that low-intensity (1.5 mT) pulsed EM fields were able to stimulate secretome activation, enhancing myogenesis with orientation-specific effects [292]. There is also considerable evidence that EM fields re-pattern tissues in wound repair, with significant regulation of inflammatory pathways, enhanced proliferation and tubulization of endothelial cells to generate new vasculature, and marked re-epithelialization with collagen deposition [209]. Indeed, regenerative therapies are being developed which involve the use of EM fields to trigger free ion release (e.g., Ca^2+^, Na^+^, K^+^) in cells and, in turn, downstream signaling cascades to proliferate and differentiate mesenchymal stem cells [293]. EM fields have already been used to upregulate the expression of osteogenic factors (e.g., BMP-2, osteopontin, COL1, and ALP) for bone regeneration with effects on several cell types [294, 295].

An emerging alternative to classic anti-cancer drugs is the use of tumor-treating fields. Non-ionizing radio frequency (RF) radiation has been used to locally heat cancerous tissues (> 45 °C) for direct ablation or sensitization to other treatments; however, because needles must be inserted directly into the tumor, applications are limited, the effect is highly localized, and the technique is invasive [296]. Alternatively, ferromagnetic nanoparticles can be injected into the tumor site and excited with kHz-to-THz-range RF pulses to ablate tumors. However, low-frequency, weak-intensity (µT) EM fields can also inhibit tumor growth by inducing calcium ion influx and, potentially, several downstream molecular targets including cyclin expression [297]. In addition to affecting cells directly, applied EM fields can change the environment around the tumor itself, including effects on ECM remodeling and modulation of inflammatory response [298]. These fields can play multiple roles, from producing an environment which is less compatible with tumor development and spread, to increasing the absorption rate of specific chemotherapeutic or radiopharmaceutical drugs [299]. Interestingly, magnetic fields have been shown to suppress the growth of some tumors [300] and promote others [301]; however, the frequency, amplitude, and pattern of the applied EMF are critical. Unlike molecular signals, EM fields are not limited to the activation of a limited set of specific receptor sub-types; rather, they can activate several parallel endogenous bioelectric currents and conserved intracellular pathways which are shared among most or all cells. Therefore, the bioelectromagnetic modality is widely applicable as a biophysical mechanism of cellular control.

### Photobiomodulation

Photobiomodulation is gaining attention as a technique to program tissue plasticity and patterning. All cells express light-sensitive molecules, from enzymes (e.g., cytochrome c oxidase) to light-gated ion channels (e.g., TRP channels), and G-protein coupled receptors (e.g., opsins) [302]. Even amino acids such as L-tryptophan can be photoactivated [303]. In many cases, biophotonic interactions are redox-mediated phenomena with the potential to initiate transcription via ROS, nitric oxide (NO), cyclic adenosine monophosphate (cAMP), and other secondary mediators [304]. It is, therefore, unsurprising that, in search of therapeutic applications, researchers have explored the effects of applied light on stem cell function including differentiation, proliferation, gene expression, cytokine release, morphogen release, metabolic function, and bioelectricity [305]. Encouragingly, photobiomodulation has demonstrated clinically relevant effects in wound healing, vascularization, tissue repair following heart attack, and other ischemic conditions, neurodegeneration, and other pathologies [306].

Healing and repair can be stimulated by photobiomodulation by triggering inflammatory activations including the release of interleukins, tumor necrosis factor (TNF), and several growth factors [307]. Infrared (830 nm), low-level laser light (50 J/cm^2^) exposures triggered significant bone regrowth and re-patterning due to activation of mitogen-activated protein kinase (MAPK) and β-catenin pathways and downstream osteogenic gene expression of BMP-4 and RUNX-2 [308]. Skin regeneration and vascularization have also been stimulated by photobiomodulation, including the activation of VEGF and pericyte mobilization [309]. Similarly, regeneration of peripheral nerves including sciatic, facial, fibular, and vagus nerve has been demonstrated using photobiomodulation within the red-to-infrared component of the EM spectrum [310]. As clinical applications have become viable, implantable devices have been engineered to deliver light-based therapies directly to tissues including uses in dentistry; however, there is a significant need to improve dose and other parameters [311]. Nevertheless, implanted photobiomodulators have been used to accelerate orthodontic movement in molar intrusions [312] and periodontal bone healing [313]. Similar implants made from biocompatible optical fibers composed of poly(L-lactic acid) produced similar effects when delivering green light to experimental femoral bone injuries in rodents [314].

While cancer cells emit light signatures that are diagnostically relevant [197, 198, 315], the impact of photobiomodulation on cancer and its therapeutic potential is less clear. A recent systematic review of 67 studies examining the effects of photobiomodulation on tumor growth, recurrence rate, proliferation, differentiation, and survival demonstrated that light-based therapies is generally safe; however, outcomes are often mixed and likely dependent on tissue-specific factors [316]. Tumor regression following light exposure has been reported in several animal models [317, 318] with proposed mechanisms including immune system activation [307]. Interestingly, photobiomodulation can sensitize tumors to irradiation, increasing tumor necrosis while protecting normal tissue [319]. It has also been used to prevent and manage toxicity and side effects associated with traditional cancer treatments including neuropathy, oral mucositis, and lymphedema [320]. However, results from photobiomodulation therapies in animal models of subcutaneous melanoma [321], anaplastic thyroid cancer [322], and a cheek pouch model of carcinogenesis [323] indicate that tumors exposed to low-intensity (< 5 W/cm^2^) light with wavelengths of approximately 660 nm may exacerbate tumor growth and contribute to poor differentiation of cytoarchitecture. Therefore, there is a timely need to identify the determinants of photobiomodulation responses and the isolation of therapeutic applications which may be used to non-invasively control tissue dynamics.

## Conclusion

Consistent with their shared molecular pathways, regenerative and cancerous tissues display common biophysical regulators of plasticity and patterning (or mispatterning). With mechanical forces, it seems that, in general, increased ECM stiffness promotes cancer cell induction, tumor growth, and metastasis while inhibiting regenerative potential in favor of scar formation. While it may be tempting to plot cancer and regeneration at opposite points on a continuum, they share several bioelectric signatures including a significantly depolarized resting membrane potential. Both systems, which involve cell migration toward different ends, are responsive to electric field gradients that guide outgrowths. Differentiation, cell division, and migration are also regulated by ambient and applied light in both regeneration and cancer. As discussed, tissue patterns associated with embryogenesis and recapitulated during regeneration may be stabilized by gap junctional networks, where disruptions result in teratogenic phenotypes or malignancies. Perhaps most clear is the previously underestimated interaction between molecular and physical modalities within the extended microenvironment, where mechanical, electrical, or electromagnetic signaling converge upon gene expression and factor release with feedbacks to modulate the environment in turn. Top-down biophysical control mechanisms are intrinsically linked to the ECM composition, structural topography, and cytoarchitecture of the microenvironment. Recognition of this is evidenced by parallel trends in regenerative medicine and oncology toward the modulation of tissue microenvironments to program physiological outcomes bypassing the micromanagement of individual cells. Our review of the literature suggests that biophysical phenomena are receiving increased attention as determinants of cell state and fate; however, there is a need to further integrate molecular and biophysical factors in consideration of their meaningful interactions.

While traditional cell culture methods provide conditions to isolate individual variables to better understand cell signaling, the interconnectedness of several molecular and biophysical signaling modalities demands the development of model systems with increased physiological relevance. Three-dimensional cell culture techniques incorporate customizable ECM structures in vitro, enabling the assessment of cell–substrate interactions which are central to biophysical signaling. Current tissue engineering applications in animal models are beginning to enable such integration. Indeed, by combining topographical alignment, chemical cues, and electric fields, it was recently demonstrated that myofibroblast could be differentiated from dermal fibroblasts and tuned to enhance wound healing with pronounced matrix reconstruction [324]. The full-circle integration of ECM-embedded cues, cell mobilization, and microenvironmental remodeling that is now possible provides a foundation for novel pro-regenerative and anti-cancer therapies.

As 3D tissue cultures have become more accessible tools, it has become possible to recapitulate the relationships between matrix geometry and bioelectricity or the impact of tissue orientation on electromagnetic field effects within tractable and scalable platforms. Novel questions can now be addressed: Are long-range bioelectric signals impacted by the polarization of ECM polymers? Can optical signaling be disrupted by ECM composition? Can tumorigenic microenvironments be remodeled by combinations of biophysical signaling patterns? In addition to enabling unprecedented experiments, 3D tissues also permit the integration of implantable materials for enhanced translation of engineered biophysical interventions. To fully harness the potential of biophysical control over tissue plasticity and patterning, 3D tissue culture systems can be used to systematically disentangle determinant factors in ways that were previously impractical when restricted to the use of animal models of cell culture with monolayers. Once the physicochemical complexities of living systems are better understood, the full spectrum of cellular communication will become available to bioengineers in search of means to medically restore or stabilize form and function in the clinical setting.

## Data Availability

None.
